# Modulating Neuro-Immune-Induced Macrophage Polarization With Topiramate Attenuates Experimental Abdominal Aortic Aneurysm

**DOI:** 10.3389/fphar.2020.565461

**Published:** 2020-08-28

**Authors:** Xing Chen, Yang Li, Jie Xiao, Hua Zhang, Chuanlei Yang, Zhanjie Wei, Weiqiang Chen, Xinling Du, Jinping Liu

**Affiliations:** ^1^Department of Cardiovascular Surgery, Union Hospital, Tongji Medical College, Huazhong University of Science and Technology, Wuhan, China; ^2^Department of Cardiovascular Surgery, Central Hospital of Wuhan, Huazhong University of Science and Technology, Wuhan, China; ^3^Department of Thyroid and Breast Surgery, Central Hospital of Wuhan, Huazhong University of Science and Technology, Wuhan, China; ^4^Department of Cardiovascular Surgery, Zhongnan Hospital, Wuhan University, Wuhan, China

**Keywords:** abdominal aortic aneurysm, topiramate, γ-aminobutyric acid, neuro-immune, macrophage polarization

## Abstract

The development of abdominal aortic aneurysm (AAA) is attributed to psychological and physical factors. Topiramate, which is an agonist of the GABA_A_ receptor, makes contributions to neuronal disease and is partially involved in immune regulation, may be effective upon abdominal aortic aneurysm progression. We used experimental abdominal aortic aneurysm models: Angiotensin II (Ang II)–induced ApoE^−/−^ male mice (Ang II/APOE model) in our study. In the Ang II/APOE model, all mice (n=64) were divided into four groups: sham group (PBS treatment), control group (Ang II treatment), low-dose group (Ang II + low-dose topiramate, 3 mg/day per mouse), and high-dose group (Ang II + high-dose topiramate, 6 mg/day per mouse). All treatments began on the day after surgery. Moreover, collected tissues and cultured cell were used for histology and biochemical examination. *In vitro*, the effects of topiramate on bone marrow-derived macrophage stimulated by LPS were investigated. Our data implied that topiramate treatment significantly promoted macrophages preservation and conversion of M1 to M2 macrophage phenotypes *in vivo* and *in vitro*. Accordingly, proinflammatory activities mediated by the M1 macrophages were decreased and the repair process mediated by M2 macrophages was enhanced. The low-dose and high-dose groups had abdominal aortic aneurysm incidences of 50% and 37.5%, respectively, compared with 75% in the control group. Topiramate, a promising drug for the psychological disease, that target neuro-immune-induced macrophage polarization may attenuate experimental abdominal aortic aneurysm progression.

## Introduction

With the rapidly growing aging population worldwide, abdominal aortic aneurysm (AAA) is becoming more prevalent in different countries and regions. Moreover, AAA has been the third most major cause of cardiovascular death, about 1% to 3% of all deaths in males >65 years old (Sakalihasan et al., 2005).

Although the incidence of AAA is on the rise, the mechanisms for the formation of aortic aneurysm remain unclear (Salmon et al., 2013; Yan et al., 2020). Except for surgery, few medical therapies can be used for confirmed AAA. Furthermore, the surgery typically is aimed at relatively large AAA rather than small or early stage AAA. As asymptomatic AAA can result in unpredictable fatal rupture (Powell and Greenhalgh, 2003), effective drugs preventing AAA dilation or protecting it from rupture are urgent to exploit.

Macrophages, which are widely related to inflammation, can be activated by chronic inflammatory states, such as atherosclerosis and oxidative stress (Liu and Jin, 2016; Wang et al., 2018). Subsequently, macrophages, including a proinflammatory M1 macrophage phenotype and an antiinflammatory M2 macrophage phenotype, infiltrate vessel walls and produce proteases like matrix metalloproteinase (MMP) and secrete inflammatory cytokines that are responsible for M1 macrophage cytokine secretions, including tumor necrosis factor (TNF)-α, interleukin-6 (IL-6), interferon-γ (IFN-γ) and interleukin-1β (IL-1β) (Kaneko et al., 2011; Murray and Wynn, 2011; Nosoudi et al., 2015). These processes further exacerbate inflammatory responses. On the contrary, M2 macrophages, releasing interleukin-10 (IL-10) and TGF-β, are considered antiinflammatory (Humeres et al., 2016; Pope et al., 2016). Particularly, recent study demonstrated that TGF-β neutralization can promote the expression of Arg-1 and aneurysmal dilation (Raffort et al., 2019). Therefore, changing the M1/M2 macrophage ratio by modulating polarization may be effective for preventing AAA expansion and rupture.

The immune system responds to injury through inflammation and the nervous systems secretion of neurotransmitters provides instantaneous homeostatic control mechanisms. These two systems interact with each other to maintain homeostasis of the ever-changing microenvironment. (Chavan et al., 2017). GABA, a classical inhibitory neurotransmitter of the central nervous system, is also detected in the peripheral nervous system including the gastrointestinal tract, pancreas and especially the endothelial cells of the vascular wall (Sen et al., 2016). Topiramate, which is a specific GABA_A_ receptor agonist, is mainly used for the treatment of partial and generalized seizures (Chung, 2015). Furthermore, GABA and GABA_A_ receptors have been reported to make a difference in modulating inflammation or the immune response (Bhat et al., 2010; Jin et al., 2013; Auteri et al., 2015). These investigations identify GABA, accompanied with the GABA_A_ receptor, as a negative inflammatory regulator in the neuro-immune interaction (Crowley et al., 2016).

However, until recently, there has been no investigation regarding the role and therapeutic potential of GABA or GABA A receptor agonists for treatment of AAA. Additionally, recent studies reported that topiramate can modulate post-infarction inflammation by converting the ratio of monocytes/macrophages subpopulation. (Wang et al., 2017). Hence, we hypothesized that agonizing the activity of the GABA_A_ receptor by using topiramate treatment could attenuate the development of experimental AAA in neuro-immune interaction. Neuro-immune dialog may generate novel insights of interest in terms of AAA clinical therapy.

## Materials and Methods

### Human Aortic Samples

We collected abdominal aortic wall specimens from five patients with AAA who underwent open surgical repair and five patients who underwent kidney transplantation, with nonaneurysmal abdominal aortic wall specimens used as controls, as previously described (Wei et al., 2014). All protocols that involved human aortic samples have been approved by the Ethical Committee of Tongji Medical College and Huazhong University of Science and Technology.

### Mice and Aneurysm Model Creation

Male apolipoprotein E-deficient (*ApoE*^−/−^) 12-week-old mice (from HFK BioTechnology Co., Ltd, Beijing) were used. All animal experiments were conducted strictly in accordance with the National Institutes of Health (NIH) Guidelines for the Care and Use of Laboratory Animals and approved by the Ethics Committee of Tongji Medical College, Huazhong University of Science and Technology. The experimental AAA model was constructed by subcutaneous Ang II infusion in *ApoE*^−/−^ mice (Ang II/APOE model) and Ang II (Sigma-Aldrich) was infused *via* subcutaneous osmotic pumps (Alzet Osmotic Pumps, model 2004, Durect Corporation) at 1,000 ng/kg/min for 28 days. Topiramate (the high-dose group: 6 mg/day per mouse, the low-dose group: 3 mg/day per mouse) or DMSO (the sham group and the control group) was intra-peritoneally injected daily from the day after the operation.

### Echocardiography for Quantification of Aneurysm Formation

Serial abdominal echocardiography was performed on 0, 7, 14, 21, and 28 days after subcutaneous Ang II infusion by a high-resolution Vevo 2100 Microimaging System (Visualsonic, Canada) in a blind manner for all mice. We selected the suprarenal aorta as the measuring point in the Ang II/APOE model. The formation of experimental AAA was defined as a ≥50% dilation of aortic diameter or the occurrence of aortic dissection (AD). At the same time, all mice were monitored daily for analysis of the survival ratio.

### Bone Marrow-Derived Macrophage Isolation and Culture

Bone marrow-derived macrophages (BMDMs) were isolated from C57BL/6 mice, as described previously (Masters et al., 2010). Specifically, mice were euthanized using cervical dislocation. The tibia and femur were free from the surrounding muscle and fat tissue, sterilized in 70% ethanol for 10 s, and then washed in PBS. With the ends of each tibia and femur removed, the bone marrow was ﬂushed out by DMEM supplemented using a 25-gauge needle. Next, cells from the bone marrow were passed through a cell strainer of 70 µm and washed with PBS after centrifugation (2,000 rpm, 3min). Then, cells were plated in a 10-cm dish at a density of about 13,000 cells/8 ml media (DMEM with 10% FBS, M-CSF at 10ng/ml, and 1% penicillin/streptomycin). With BMDMs grown at 37˚C for 7 days, the media was removed and replaced with the same fresh media every 2 days. Flow cytometry showed the purity of macrophages about 98% at 1 week. At the 7^th^ day, BMDMs were treated for 6 h with different treatments: control (no treatment); LPS group (LPS 100 ng/ml); topiramate group (LPS 100 ng/ml and topiramate 200 µM); IL-4 group (IL-4 10 ng/ml).

### Flow Cytometry

The proportion of macrophage in the spleen of mice or BMDMs were analyzed by flow cytometry. These cells were stained with APC-CY7-anti-CD45, Fitc-anti-CD11b, PE-anti-F4/80, PE-CY7-anti-CD86, APC-anti-CD206, and their isotype controls (eBioscience, CA) according to the protocol. Flow cytometry was performed with the FACSCalibur (BD Immunocytometry Systems). All data were analyzed using FlowJo software.

### RNA Isolation and Real-Time PCR (RT-PCR)

Total RNA was extracted from cultured cells with Trizol reagent (Takara Bio, Tokyo, Japan) according to the manufacturer’s protocols. RNA was reverse-transcribed using the RNA PCR Kit (Takara Bio, Tokyo, Japan). Quantitative polymerase chain reaction (qPCR) ampliﬁcation using SYBR Green PCR Master Mix (Takara Bio, Tokyo, Japan) was performed on an ABI PRISM 7900 Sequence Detector system (Applied Biosystem, Foster City, CA). Relative gene expression was calculated by the method of 2^−ΔΔCt^. The RT-PCR primer sequences are shown in the following. The 18 s was used as the endogenous control.

QRT-PCR primers used.

**Table:** 

Gene	Forward primer	Reverse primer
Arg-1 iNOS Ym-1 CD206 18s	TTTAGGGTTACGGCCGGTG TCCTGGACATTACGACCCCT GGGCCCTTATTGAGAGGAGC TTGCACTTTGAGGGAAGCGA TTGACGGAAGGGCACCACCAG	TCCTCGAGGCTGTCCTTTTG CTCTGAGGGCTGACACAAGG TGAGAGCAAGAAACAAGCATGG CCTTGCCTGATGCCAGGTTA GCACCACCACCCACGGAATCG

### Western Blot

BMDMs or tissue taken from the aorta of mice were harvested in ice-cold lysis buffer containing 4% proteinase inhibitor (Sigma-Aldrich). Protein was quantified by the BCA protein assay kit (Thermo Fisher Scientific, USA). Equal proteins were separated on SDS polyacrylamide mini-gels and transferred to the PVDF membrane. This was followed by washing with Tris-buffered saline (TBS) and 5% nonfat dry milk for blocking, and was incubated overnight with primary antibodies: MMP9 (diluted at 1:1,000, ab38898, Abcam), TNF-α (1:1,000, ab6671, Abcam), IL-1β (diluted at 1:5,000, ab9722, Abcam), IL-6 (diluted at 1:1,000, ab7737, Abcam), phosphor-NF-κB p65 (diluted at 1:1000, 3039S, CST), NF-κB p65 (diluted at 1:1,000, 8242T, CST), phospho-IκBα (diluted at 1:500, 5209S, CST), IκBα (diluted at 1:500, 4812S, CST), GAPDH (diluted at 1:1,000, 5174S, CST) and β-actin (diluted at 1:1,000, ab8227, Abcam). Membranes were washed and incubated with peroxidase-conjugated secondary antibody for 1 h at room temperature. Bands using the ECL kit were detected with a Bio-Rad (Hercules, CA) imaging system.

### Immunofluorescence and Immunohistochemical Staining

After mice were euthanized, the abdominal aorta was carefully separated (avoiding the vessel’s injury) and was ﬁxed in 4% paraformaldehyde for immunofluorescence and immunohistochemistry. The primary antibodies used in immunofluorescence and immunohistochemistry were MCP-1 (Rabbit polyclonal, diluted at 1:200, ab9669, Abcam), iNOS (Rabbit polyclonal, diluted at 1:200, ab3523, Abcam), Arg-1 (Rabbit polyclonal, diluted at 1:200, ab91279, Abcam), MMP9 (Rabbit polyclonal, diluted at 1:500, ab38898, Abcam), TNF-α (Rabbit polyclonal, diluted at 1:200, ab6671, Abcam), IL-1β (Rabbit polyclonal, diluted at 1:200, ab9722, Abcam), and IL-6 (Rabbit polyclonal, diluted at 1:200, ab7737, Abcam). The nuclei were stained by DAPI.

### Statistical Analysis

All data are presented as mean ± SEM. Unpaired, two-tailed Student t-test was used to analyze normally distributed data from two groups. Multiple comparisons were analyzed by one-way analysis of variance (ANOVA), followed by Tukey’s post-test. Differences with P<0.05 were considered statistically significant. All the experiments were repeated for at least three replicates per condition.

## Results

### M1 Accumulation and Increased Expression of IL-1β, TNF-α, and MCP-1 in Human AAAs

**Figure 1:**
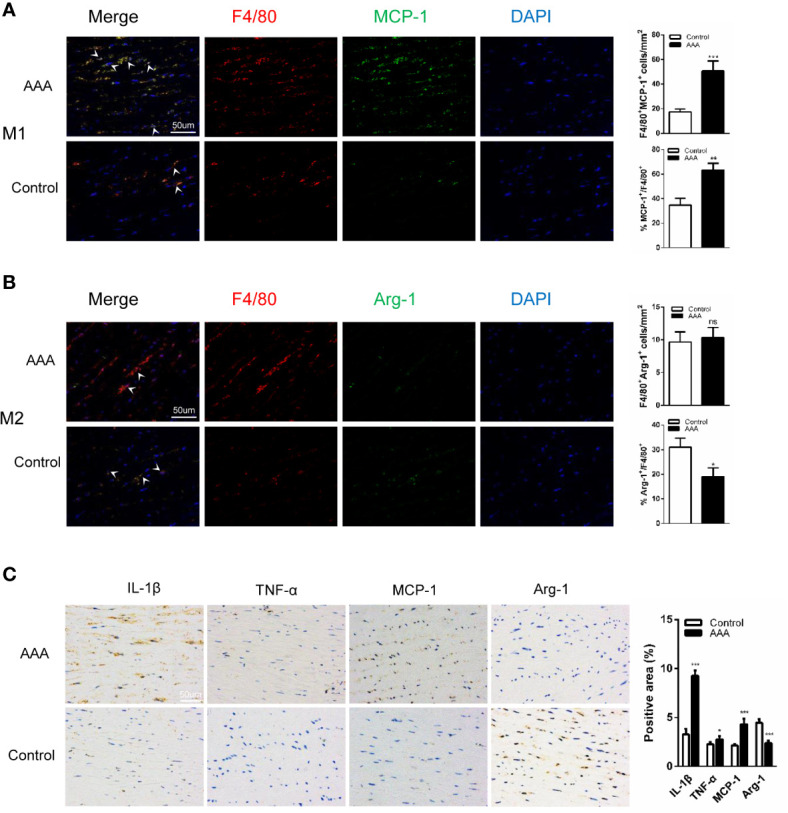
M1 accumulation and increased expression of interleukin-1β (IL-1β), tumor necrosis factor–α (TNF-α), and MCP-1 in human AAAs. **(A**, **B)** Immunofluorescence staining of (F4/80; red), (MCP-1; green) and (Arg-1; green) in the aortic tissues from AAA patient and donor (Control). (n=5 per group. scale bars =50 µm). **(C)** Immunohistochemical staining of IL-1β, MCP-1, Arg-1, and TNF-α. (n=5 per group. scale bars =50 µm). Quantification of five randomly selected fields of view per sample. *P<0.05, **P<0.01, ***P<0.001 versus control group. Ns indicates no significance. Scale bars = 50 µm.

To fully investigate the role of GABAergic drugs during AAA progression, we first examined the human tissues. In addition to AAA specimens, normal aortic tissues were collected and used as the control group. We analyzed all samples by Immuno-histochemical and Immunofluorescence staining. Compared with the control specimens, macrophages especially M1 macrophages (F4/80^+^MCP-1^+^ cells) were much more accumulated in aortic tissue samples of AAA patients. For M2 macrophages (F4/80^+^Arg-1^+^ cells), there was no significant difference in the control specimens and aortic tissue samples of AAA patients (**Figures 1A, B**). In addition, proaneurysmal molecules, like IL-1β, TNF-α, and MCP-1, were significantly increased in human AAA sections by immune-histochemical staining (**Figure 1C**). Of course, to assess the grade of structural damage, it was also important that the SMC destruction and the median elastin scored on a histology grading scale from mild (I) to severe (IV). In the AAA group, scores were significantly increased (**Supplemental Figure 1**). These results demonstrate a drastic pathological progression and implied that proinflammatory M1 macrophage activities are involved in the formation of human AAA.

### A Decrease in Serum GABA Accompanies AAA Formation

**Figure 2:**
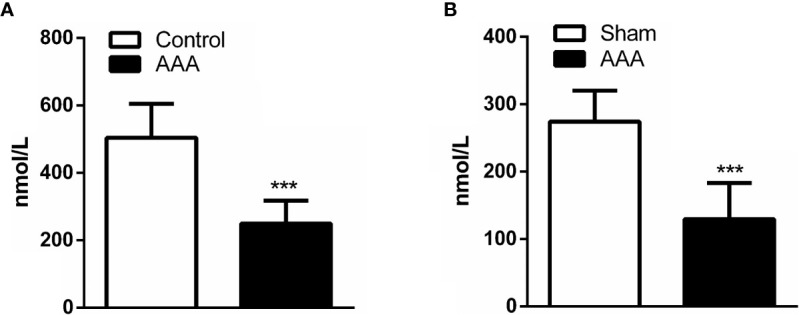
A decrease in serum GABA accompanies abdominal aortic aneurysm (AAA) formation. **(A)** The concentration of GABA in the peripheral blood is lower in AAA patients compared with normal people (Control) (n=5 per group). **(B)** The serum concentration of GABA reduce in Ang II-induced AAA model (AAA) respective with *ApoE*^−/−^ mice (Sham) by ELISA (n=5 per group). ***P<0.001.

Previous investigations found serum GABA in human at a concentration between 0.5 and 3 μmol/L. Here, we examined serum GABA from the people without AAA and AAA patients by ELISA. We found that the levels of serum GABA in AAA patients reduced significantly compared with the people without AAA (**Figure 2A**). Meanwhile, we detected the concentration of GABA in the peripheral blood of ApoE^−/−^ mice and found that the levels of serum GABA in the Angiotensin II/ApoE^−/−^ models were lower than that in the sample ApoE^−/−^ mice (**Figure 2B**).

### Treatment With Topiramate Reduces the AAA Incidence and Increases Murine Survivals in the Ang II/APOE Model

**Figure 3:**
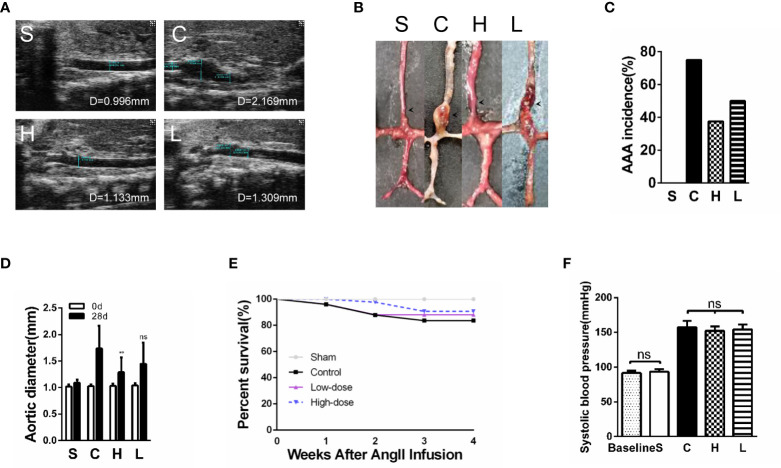
Treatment with topiramate reduces the abdominal aortic aneurysm (AAA) incidence and prolongs murine survivals in the Ang II/apolipoprotein E-deficient (APOE) model. **(A)** Representative ultrasound images of the abdominal aorta cross-sectional tissue from each group on days 28 after Ang II infusion (n=16 in Sham group, n=10 in Control group, n=13 in High-dose group, n=12 in Low-dose group). **(B)** Quintessential photographs showed the obvious changes in the Ang II-induced AAA model (indicated by the arrows). **(C)** The incidence of the AAA. **(D)** A graph of the maximum diameter of each group (n=16 in Sham group, n=10 in Control group, n=13 in High-dose group, n=12 in Low-dose group). **(E)** Survival of mice in each group. **(F)** Systolic blood pressure of mice in each group (n=16 in Sham group, n=10 in Control group, n=13 in High-dose group, n=12 in Low-dose group). **P<0.01 vs. the control group. Ns indicates no significance. S=Sham, C=Control, H=High-dose, L=Low-dose.

As reported previously, the experimental AAA, which was defined as a ≥50% increase in the aortic diameter or the occurrence of an aortic dissection, was successfully induced in our experiments. The aortic diameter (luminal diameter) was measured by ultrasonography on the 28^th^ day after Ang II infusion. By analyzing the ultrasound results and digital photographs of the aorta, we found that the AAA diameter of mice with high-dose topiramate treatment was significantly smaller than the control group, but there was no statistical significance between the control group and the low-dose group (**Figures 3A, B, D**). AAA incidence had a dose-dependent decline after topiramate treatment. In the high-dose group (6 mg/day per mouse), the AAA incidence was significantly reduced from 75% (control group, 12 of 16) to 37.5% (6 of 16). The AAA incidence in the low-dose group (3 mg/day per mouse) was higher than that in the high-dose group (50%, 8 of 16) (**Figure 3C**). In accordance to the reduced AAA incidence, high-dose topiramate treatment elevated the survival ratio from 62.5% to 81.25% compared with the control group (**Figure 3E**). Though an obviously elevated trend existed, there was no statistically significant difference between the control group and the high-dose group (χ^2 =^ 1.908, P=0.1672). These results indicate that topiramate effectively ameliorates AngII-induced AAA formation in mice. Studies have shown topiramate lowed blood pressure in some degree. But we measured blood pressure of mice and found that there was no significant difference between the control group and the topiramate-treatment groups (**Figure 3F**).

### Topiramate Causes a Macrophage Subset Skewing Toward the M2 Macrophage Phenotype in the Experimental AAA Model

To assess the effects of topiramate on the macrophages and the macrophage phenotype conversion in the experimental AAA model, we analyzed M0, M1, and M2 macrophage subpopulations/percentages in the experimental AAA model. In our previous study, we found that the spleen may be involved in the progression of AAAs in the Ang II/APOE model (Wei et al., 2014). What’s more, studies have shown that Ang II could drive monocyte mobilization from the spleen and recruitment to the abdominal aorta in Ang II/APOE model (Mellak et al., 2015). Therefore, we assessed macrophages in the aortic wall as well as in the spleen. Firstly, we used hematoxylin and eosin (HE) staining and immunohistochemical staining of CD68 to detect the total macrophages of the aortic wall as well as specific macrophage markers (CD45^+^F4/80^+^CD11b^+^) by FCM to detect the splenic macrophages in the experimental AAA model. However, the data yielded a surprising outcome: there was no significant difference between the control group and the topiramate treatment group (**Supplemental Figures 2** and **3**).

**Figure 4:**
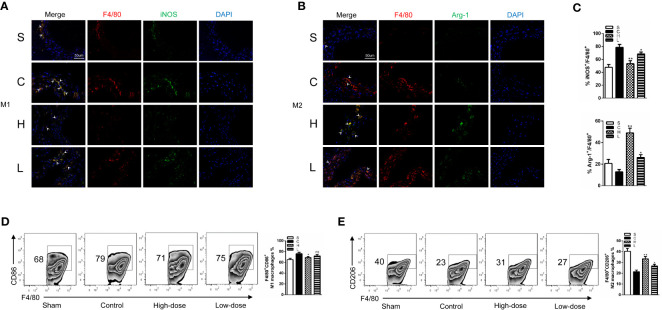
Topiramate causes a macrophage subset skewing toward the M2 macrophage phenotype in the experimental abdominal aortic aneurysm (AAA) model. **(A**, **B)** Immunofluorescence staining of (F4/80; red), (iNOS; green) and (Arg-1; green) in the cross-sectional aortic tissues from each group (**A**; M1, F4/80^+^iNOS^+^) (**B**; M2, F4/80^+^Arg-1^+^) (n=3 per group). **(C)** The ratio of M1 or M2 covered F4/80+ cells in the **(A**, **B)**. **(D**, **E)** Representative graphs of CD45^+^F4/80^+^CD11b^+^CD86^+^ M1 macrophages **(D)**, CD45^+^F4/80^+^CD11b^+^CD206^+^ M2 macrophages **(E)** in the spleen by flow cytometric (FCM) analysis (n=3 per group). Scale bars = 50 µm. *P<0.05, **P<0.01 versus control group. Ns indicates no significance. S=Sham, C=Control, H=High-dose, L=Low-dose.

Subsequently, we performed immunofluorescence staining for the polarized macrophage population with specific antibodies for M1 (F4/80^+^iNOS^+^) and M2 (F4/80^+^Arg-1^+^) population. In all four groups, there were few macrophages in the sham group and the high-dose group. Meanwhile, we found that the proportion of M1 macrophages were significantly increased in the control group; however, it was notably decreased after topiramate treatment, especially in the high-dose group. Compared with the control group, topiramate treatment gave a rise to a significant decrease in the M1 macrophage subpopulation/percentage and a markedly increase of the M2 macrophage subpopulation/percentage (**Figures 4A, B, C**). Then, we used flow cytometry (FCM) to quantify the number of M1 and M2 macrophages. In parallel with the results of immunofluorescence staining, topiramate resulted in a marked reduction of M1 macrophages and a relatively greater increase in M2 macrophages in the spleen (**Figures 4D, E**). Therefore, topiramate resulted in an M1 macrophage skew toward M2 macrophage.

### Topiramate Decreases Proinflammatory M1 Macrophage Activities in the Experimental AAA Model

**Figure 5:**
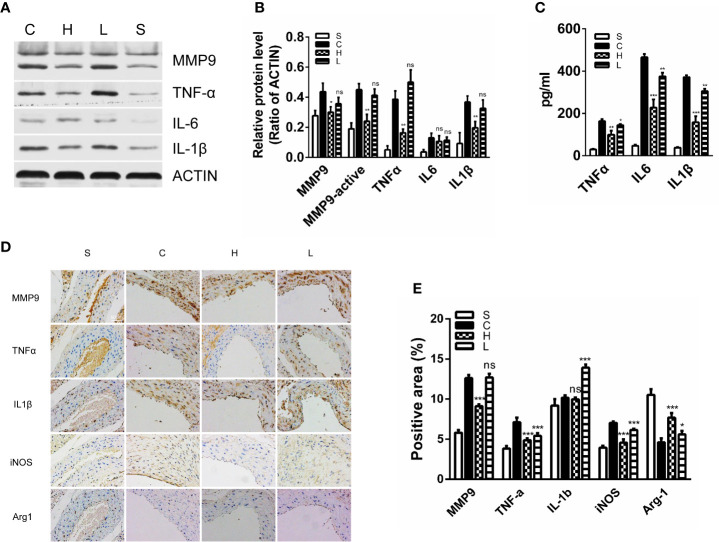
Topiramate decreases proinflammatory M1 macrophage activities in the experimental abdominal aortic aneurysm (AAA) model. **(A**, **B)** Matrix metalloproteinase 9 (MMP9), tumor necrosis factor–α (TNF-α), interleukin-6 (IL-6), and IL-1β were significantly decreased in the high-dose topiramate group compared with the control group by western blot analysis (n=3 per group). **(C)** Topiramate attenuated the release of TNF-α, IL-6, and IL-1β by ELISA (n=5 per group). **(D**, **E)** Immunohistochemical staining of MMP9, IL-1β, MCP-1, Arg-1, and TNF-α in cross-sectional aortic tissues from each group (n=3 per group). Scale bars = 50 µm. *P<0.05, **P<0.01, ***P<0.001 versus control group. Ns indicates no significance. S=Sham, C=Control, H=High-dose, L=Low-dose.

By detecting the expression of inflammatory cytokines after AngII infusion, we could assess the effects of topiramate on M1 macrophage activities. In line with the decreased trend of M1 macrophage ratio, high-dose topiramate significantly down-regulated the expression of tumor necrosis factor-α (TNF-α), IL-1β and matrix metalloproteinase (MMP)-9 as well as decreased MMP-9 activity, which contributes to the development of AAA formation. However, there was no statistical significance between the low-dose group and the control group (**Figures 5A, B**). Furthermore, we also found that topiramate regardless of the high-dose group or the low-dose group markedly decreased the levels of MMP9, IL-1β, TNF-α, and iNOS in the peripheral blood by ELISA (**Figure 5C**). In addition, proaneurysmal molecules, IL-1β, TNF-α and MCP-1 as examples, were significantly decreased by topiramate in cross-sectional aortic tissue samples of the AngII/APOE model by immunohistochemical staining (**Figure 5D, E**).

### Topiramate Alters the Proinflammatory Phenotype of LPS-Stimulated Bone Marrow-Derived Macrophages *In Vitro*

To further investigate the role of topiramate, we performed studies using bone marrow-derived macrophages (BMDMs) *in vitro* and the purity of BMDMs were about 98% (**Supplemental Figure 4**). As the AAA models were induced by Ang II *in vivo*, we first sought to evaluate whether topiramate could influence the BMDMs polarization with Ang II treatment *in vitro*. However, the RT-PCR results showed that topiramate had no significant effects on mRNA levels of iNOS and Arg-1 (**Supplemental Figure 5**).

**Figure 6:**
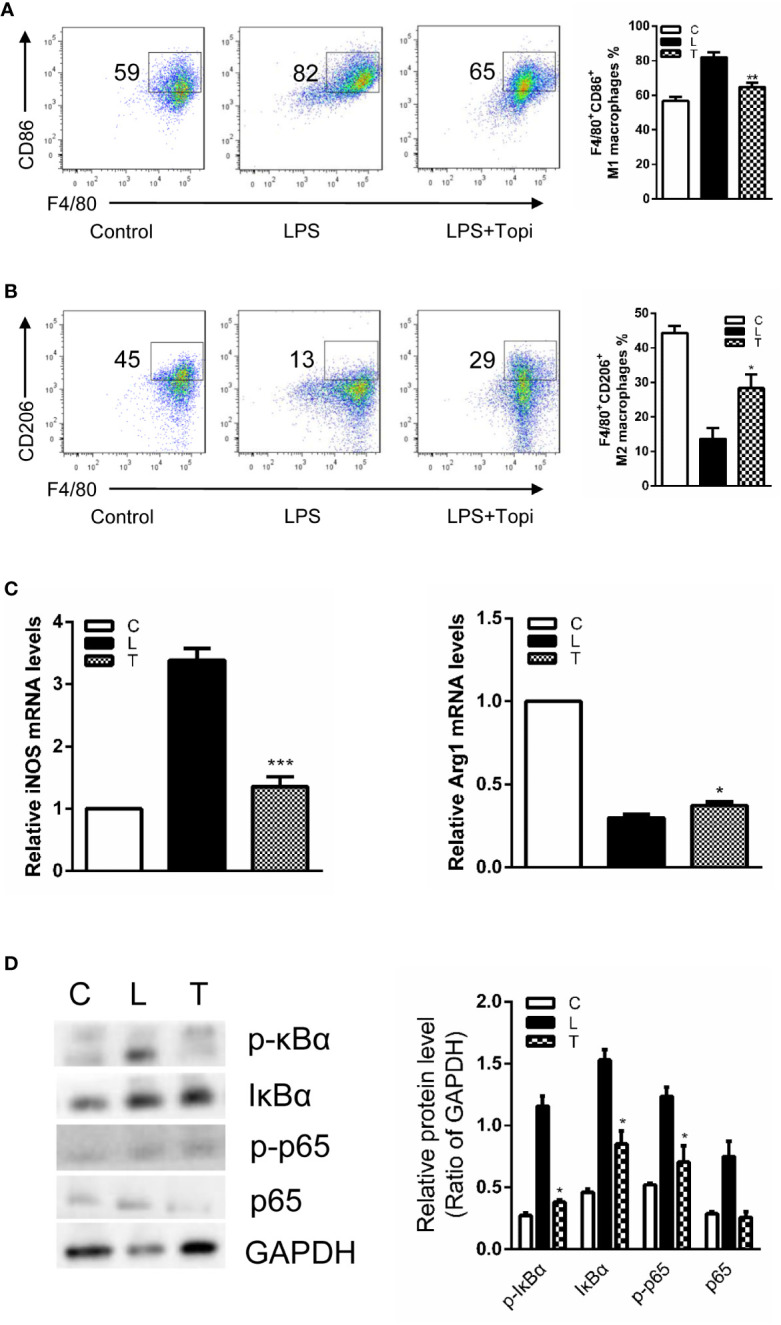
Topiramate alters the proinflammatory phenotype of lipopolysaccharide (LPS)-stimulated bone marrow-derived macrophages *in vitro*. **(A**, **B)** Flow cytometric (FCM) analysis the percent of CD45^+^F4/80^+^CD11b^+^CD86^+^ M1 macrophages **(A)** and CD45^+^F4/80^+^CD11b^+^CD206^+^ M2 macrophages **(B)** from the bone marrow. **(C)** The expression level of iNOS and Arg-1 were quantitative by real-time polymerase chain reaction (qRT-PCR). **(D)** Topiramate can decrease IκB, p-IκB and p-p65 in the LPS-induced bone marrow-derived macrophages (BMDMs) by western blot analysis. n=3 separate experiments using distinct cell isolates. *P<0.05, **P<0.01, ***P<0.001 versus LPS-induced group. C=Control, L=LPS, T=LPS + Topiramate.

Subsequently, considering the lipopolysaccharide (LPS) acting as a switch for macrophage activation, BMDMs were treated with LPS together with and without topiramate for 6 h. We found that topiramate modulate M1/M2 macrophage polarization. Compared with the LPS-induced group, topiramate triggered an increase of the M2 macrophage and a drastic decline of M1 macrophage (**Figures 6A, B**). Similarly, topiramate significantly inhibited the mRNA levels of iNOS (M1 marker) and promoted the mRNA levels of Arg-1 (M2 marker) (**Figure 6C**). In contrast to LPS, IL-4 alternatively activates macrophages and increases expression of Ym1, Arg1, and CD206. With topiramate, levels of these markers are markedly enhanced. (**Supplemental Figure 6**). Furthermore, we explored the effects of topiramate on NF-κB in modulating the macrophage polarization and topiramate was shown to inhibit the levels of IκB and p65 *in vitro* (**Figure 6D**).

## Discussion

In this work, we confirm that macrophages, especially M1 macrophages, were significantly elevated in the aortic wall of AAA; meanwhile, intriguingly, we found that the concentration of serum GABA was reduced in AAA patients compared with people without AAA. GABA is a paramount immunomodulatory molecule, and its signaling regulates the effects of some immune cells (Bhandage et al., 2018; Januzi et al., 2018). Topiramate, which is a specific GABA_A_ receptor agonist, has been noted to be associated with macrophage polarization (Wang et al., 2017). The pharmacokinetics of topiramate are linear pharmacokinetics (the dose range is 100–800mg), low oral clearance (about 22–36 ml/min), and a long half-life (about 19–25 h). Thus, we hypothesized that agonizing the activity of GABA_A_ receptor *via* topiramate treatment could attenuate the progression of experimental AAA in neuro-immune interactions.

AAA is a degenerative disease associated with genetic and environmental factors. The confirmed environmental factors include aging, the male gender, hypertension and atherosclerosis. In this study, we found that the circulating concentration of GABA was lower in AAA patients than in people without this disease. GABA is an important inhibitory neurotransmitter and it is involved in several disease states. When the body lacks GABA, it results in anxiety, fatigue and other emotions. Furthermore, many studies have found that GABA is closely related to blood pressure management and immune regulation (Kawabe et al., 2013; Ren et al., 2016). Previous investigation confirmed that mental stress, anxiety, and depression increase the risk of cardiovascular disease. (Roest et al., 2010; Steptoe and Kivimaki, 2012). Interestingly, a UK-based cohort study confirmed that distress was associated with coronary heart disease, and other cardiovascular diseases, but psychological distress was weakly related to AAA (Batty et al., 2014). Nevertheless, the pathology of AAA is complex, and inflammation plays an extremely important role in it. AAA is sometimes called inflammatory AAA. Considering the multiple complex relationships between GABA and the factors affecting the formation of AAA, such as hypertension, inflammation as well as mental and psychological conditions. Hence, the finding that a decreased circulating concentration of GABA is associated with AAA formation is of great significance.

GABA is widely distributed in humans and mammals, not only in the brain, but also in the peripheral tissues. Earlier research reported that GABA was present in lower concentrations in the cerebrospinal fluid than in the plasma. (Löscher, 1979) In a rabbit model of hepatic encephalopathy, GABA was derived from the gut and the development of hepatic encephalopathy was associated with elevated levels of GABA in plasma (Schafer and Anthony Jones, 1982). From our experimental AAA model, the development of AAA was associated with reduced levels of circulating GABA. Several studies suggested that both endocrine cells and exocrine cells, such as adrenal chromaffin cells, pancreatic B cells, gastric mucosal epithelium cells, contain GABA (Shdor and Joachim, 1990). Recently investigators examined that circulating GABA was synthesized and released by endothelial cells (Sen et al., 2016).

GABA receptors, including the ionotropic GABA_A_, metabotropic GABA_B_ and GABA_C_ receptor, is key to GABA signaling. The GABA_A_ receptor, characterized by high levels of structural diversity, have nineteen subunit isoforms, which are grouped into eight subfamilies (α1–6), β(1–3), γ(1–3), δ, ϵ, θ, π, and ρ(1–3) (Bjurstom et al., 2008). GABA_A_ receptors are predominantly expressed in the central nervous system. Synaptic and extrasynaptic GABA_A_ receptors are the most prominent players in neuronal inhibition (Sigel and Ernst, 2018). Meanwhile, peripheral tissues like the gastrointestinal tract and respiratory tract also express some GABA_A_ receptor (Auteri et al., 2015). Compared with the GABA_A_ receptor, GABA_B_ receptors only have two subunit isoforms. Presynaptic GABA_B_ receptors suppress Ca2^+^ inﬂux by inhibiting Ca^2+^ channels and postsynaptic GABA_B_ receptors triggering the opening of K^+^ channels induce inhibitory postsynaptic potentials (Gassmann and Bettler, 2012). Unlike GABA_A_ and GABA_B_ receptors, GABA_C_ receptors are a group of bicuculline- and baclofen-insensitive ionotropic GABA receptors (Zhang et al., 2001). Given the complexity and diversity of GABA and the GABA receptor, particularly considering the role of the GABA_B_ receptor as the Ca^2+^ channels inhibitor, topiramate, which is a GABA_A_-ergic drug, has been chosen to be used in our study.

In recent years, growing evidence demonstrates that GABA plays a pivotal role in the immune system (Knabl et al., 2008; Jin et al., 2013). Immune cells like lymphocytes and macrophages express the components of the GABA neurotransmitter system. GABA signaling has a significant influence on the functions of immune cells. GABA negatively regulates inflammation in neuro-immune interactions because of its impacts on the production of proinflammatory cytokines and its activation of some signal pathways, including nuclear factor-kappaB and mitogen-activated protein kinase pathways (Fukuchi et al., 2014). Macrophages are known as microglia in the central nervous system. They are an important member of immune cells and express the GABA_A_ receptor. When bound to the GABAA receptor on macrophages, GABA functions to reduce relative pre-inflammatory cytokine production (Reyes-Garcia et al., 2007). Prior study has suggested that topiramate can inhibit the formation of human macrophage-derived foam cells (Yang et al., 2014). In mouse pulmonary macrophages, during INFγ-induced M1-like activation, GABA signaling is down-regulated; with the GABA_A_ receptor agonist, these M1-like responses are significantly prevented (Januzi et al., 2018). Recent research demonstrated that GABA_A_ receptor agonist modulates post-infarction inflammation by changing the ratio of M1/M2 (Wang et al., 2017). But Wang et al. found that topiramate failed to affect either M1 or M2 macrophage polarization directly. This may be related to different derived macrophages and different intervention. Here, our investigation confirmed that GABA_A_ receptor agonist modulate macrophage polarization in the Ang II/APOE model.

However, selective M1 and M2 stimuli cannot occur separately *in vivo* and the effects of macrophage subsets on AAA are complex. Daugherty et al. reported the presence of macrophages in the aneurysmal aortic wall in animal models of AAA (Daugherty et al., 2000). Dale et al. found M2 phenotype injected into mice could reduce aortic dilation in an experimental AAA model (Dale et al., 2016). To explore the accumulation of macrophages in different aortic layers, Dutertre et al. use the flow cytometry to analyze the composition of human aneurysmal tissues (Dutertre et al., 2014). Subsequently, some researchers found a higher cellular density for M2 macrophages in the aortic wall and elevating the expression of Arg-1 (M2 marker) can promote aneurysmal dilation (Boytard et al., 2013; Raffort et al., 2019). These studies reported that the M1 subset was predominant in the adventitia, while M2 macrophages concentrated distribution in intraluminal thrombus. Besides, it is possible that macrophage infiltration and polarization are involved in various steps of AAA development and complications, and the samples collected just represented partially different stages of the disease. Meanwhile, the investigators confirmed that M1 macrophages accumulated in the early stage and prolonged infusion for additional 56 days led to a higher ratio of M2/M1 in Ang II/APOE model. The author found that TGF-β activity can protect against inflammatory aortic aneurysm progression in the model of angiotensin II-infused mice (Wang et al., 2010). In this study, we confirmed that promoting M1 macrophage skewing toward M2 macrophage could attenuate AAA progression.

Our present research corroborates the idea that agonizing the activity of GABA_A_ receptors with topiramate could attenuate the progression of Ang II/APOE model. AAA can result in unpredictable fatal rupture, but there are no effective drugs preventing AAA dilation or protecting it from rupture. The AngII-induced AAA mouse model can recapitulate the significant features of human AAA, like marked inflammation and aortic rupture. In this study, we found the higher dose of topiramate can decrease proinflammatory M1 macrophage activities and prevent the rupture of AAA in the experimental AAA. Thus, topiramate may be as a potential drug therapy for AAA. However, there are still some questions remaining unresolved. Firstly, the occurrence and development of AAA involves complicated pathophysiology, not only inflammation or macrophage infiltration. Hence, we should further investigate other possible roles that topiramate is involved in. Secondly, neuro to immune or GABA to macrophage involves a series of molecular mechanisms, such as the release of inflammatory mediators and the ion channel oligomerization and depolarization. The importance of associated signaling pathways, such as nuclear factor-kappaB and mitogen-activated protein kinase pathways, should be further addressed.

In summary, topiramate ameliorated AAA formation in AngII/APOE models, and an *in vitro* experiment manifested that topiramate can modulate macrophage polarization. Neuro-immune interaction may be a novel therapeutic approach for AAA treatment.

## Data Availability Statement

All datasets presented in this study are included in the article/**Supplementary Material**.

## Ethics Statement

The studies involving human participants were reviewed and approved by the Ethics Committee of Tongji Medical College, Huazhong University of Science and Technology. The patients/participants provided their written informed consent to participate in this study. The animal study was reviewed and approved by the Ethics Committee of Tongji Medical College, Huazhong University of Science and Technology.

## Author Contributions

XC: Methodology, investigation, writing-original draft preparation. YL: Methodology, validation, data curation. JX: Data curation, formal analysis. HZ: Visualization, software. ZW: Formal analysis. CY: Software. WC: Visualization. XD: Validation. JL: Conceptualization, supervision.

## Funding

The work was supported by National Natural Science Foundation of China (grant numbers 81570427, 81600366 and 81974039).

## Conflict of Interest

The authors declare that the research was conducted in the absence of any commercial or financial relationships that could be construed as a potential conflict of interest.

## Abbreviations

AAA, abdominal aortic aneurysm; AngII, AngiotensinII; *ApoE*^-/-^, apolipoprotein E-deficient; BMDMs, bone marrow-derived macrophages; FCM, flow cytometry; GABA, γ-aminobutyric acid; H&E, hematoxylin and eosin; IFN-γ, interferon-γ; IL-1β, interleukin-1β; IL-10, interleukin-10; IL-6, interleukin-6; MMP, matrix metalloproteinase; MMP-9, matrix metalloproteinase-9; TNF-α, tumor necrosis factor α.
