# Supplementary material for: Modulating Neuro-Immune-Induced Macrophage Polarization With Topiramate Attenuates Experimental Abdominal Aortic Aneurysm
Source: Front Pharmacol. 2020 Aug 28;11:565461. doi: 10.3389/fphar.2020.565461 (PMC7485436; doi:10.3389/fphar.2020.565461)
Supplement: Supplementary file 1 [file DataSheet_1.zip › Supplemental material/supplemental material.docx]

**Modulating Neuro-immune-induced Macrophage Polarization with Topiramate Attenuates Experimental Abdominal Aortic Aneurysm**

Xing Chen^1*^, Yang Li^1*^, Jie Xiao^2^, Hua Zhang^1^, Chuanlei Yang^2^, Zhanjie Wei^3^, Weiqiang Chen^1^, Xinling Du^1#^, Jinping Liu^4#^

1 Department of Cardiovascular Surgery, Union Hospital, Tongji Medical College, Huazhong University of Science and Technology, Wuhan, China

2 Department of Cardiovascular Surgery, Central Hospital of Wuhan, Huazhong University of Science and Technology, Wuhan, China

3 Department of Thyroid and Breast Surgery, Central Hospital of Wuhan, Huazhong University of Science and Technology, Wuhan, China

4 Department of Cardiovascular Surgery, Zhongnan Hospital, Wuhan University, Wuhan, China

^#^Corresponding author: Jinping Liu, Ph.D. M.D. Department of Cardiovascular Surgery, Zhongnan Hospital, Wuhan University, Wuhan, 430071, China

Tel/Fax: 86-13986127672

E-mail: [jinping@hust.edu.cn](mailto:jinping@hust.edu.cn).

^#^Corresponding author: Xinling Du, Ph.D. M.D. Department of Cardiovascular Surgery, Union Hospital, Tongji Medical College, Huazhong University of Science and Technology, Wuhan, 430022, China.

Tel/Fax: 86-27-85351609

E-mail: 1518205226@qq.com.

^*^These authors contributed equally to this work.

**Supplemental Figure 1.** Elastin fibers shown with Elastic van Gieson (EVG) stain and elastin destruction score. (n=5 per group). ***P<0.001 versus control group. Scale bars = 50 um.

**Supplemental Figure 2.** Hematoxylin and eosin (HE) and immunohistochemical staining of CD68 in cross-sectional aortic tissues from each group to detect the macrophages infiltration of the aortic wall and there was no significant difference between the control and the topiramate treatment group (n=4-7 per group). Scale bars = 50 or 100 um.

**Supplemental Figure 3.** Flow cytometry (FCM) with specific macrophage markers (CD45^+^F4/80^+^CD11b^+^) to detect the macrophages of the spleen (n=5 per group).

**Supplemental Figure 4.** Flow cytometry showed the purity of macrophages about 98% at 1 week. n=3 separate experiments using distinct cell isolates.

**Supplemental Figure 5.** The expression level of iNOS and Arg-1 were quantitative by real-time polymerase chain reaction (RT-PCR). Ns indicates no significance. Topi=Topiramate. n=3 separate experiments using distinct cell isolates.

**Supplemental Figure 6.** The expression level of Ym-1, Arg-1, and CD206 were quantitative by real-time polymerase chain reaction (RT-PCR). n=3 separate experiments using distinct cell isolates. **P<0.01. Topi=Topiramate.
