# Supplementary figures and images for: Modulating Neuro-Immune-Induced Macrophage Polarization With Topiramate Attenuates Experimental Abdominal Aortic Aneurysm
Source: Front Pharmacol. 2020 Aug 28;11:565461. doi: 10.3389/fphar.2020.565461 (PMC7485436; doi:10.3389/fphar.2020.565461)

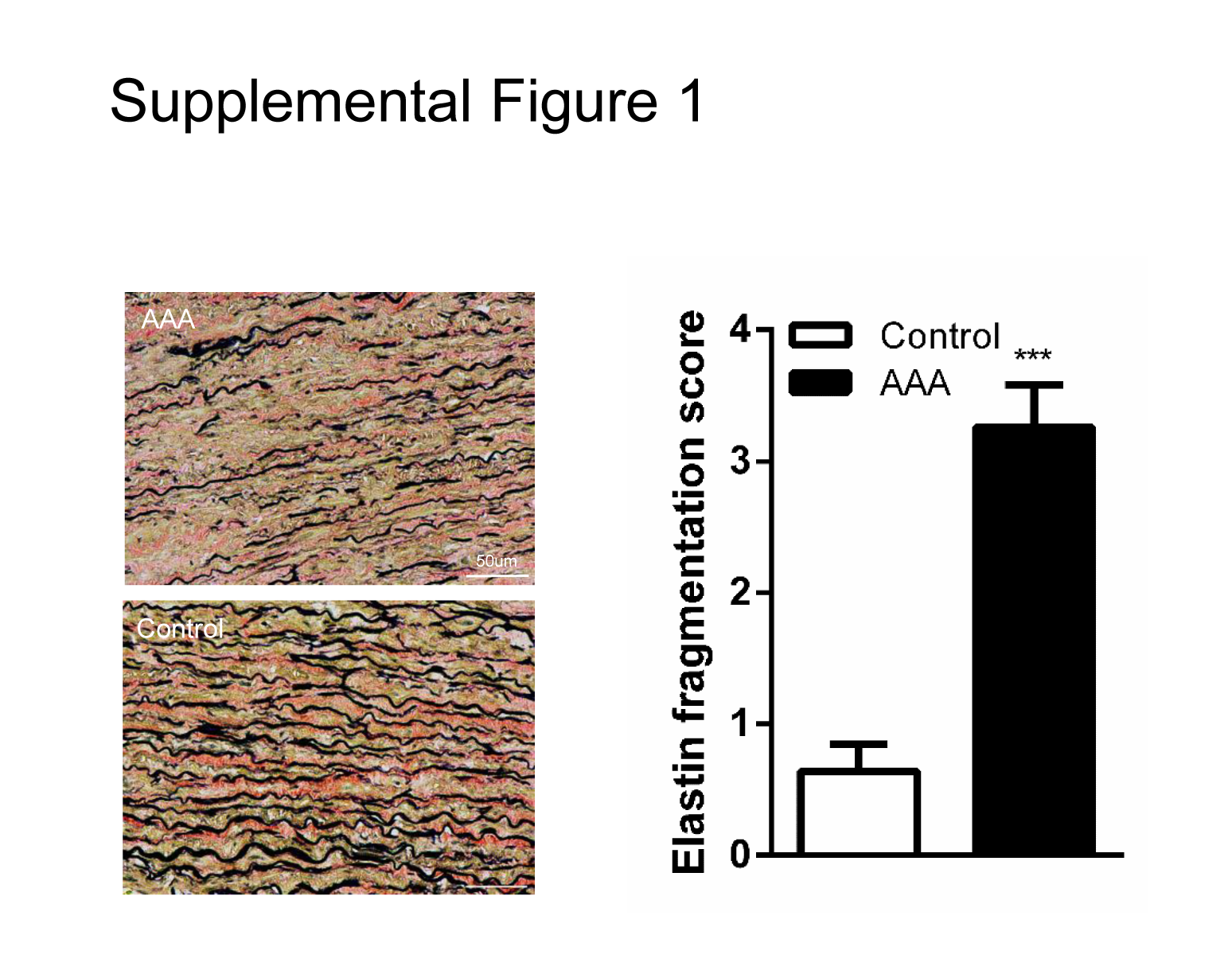

Supplement: Supplementary file 1 [file DataSheet_1.zip › Supplemental material/Supplemental Figure 1.tif]

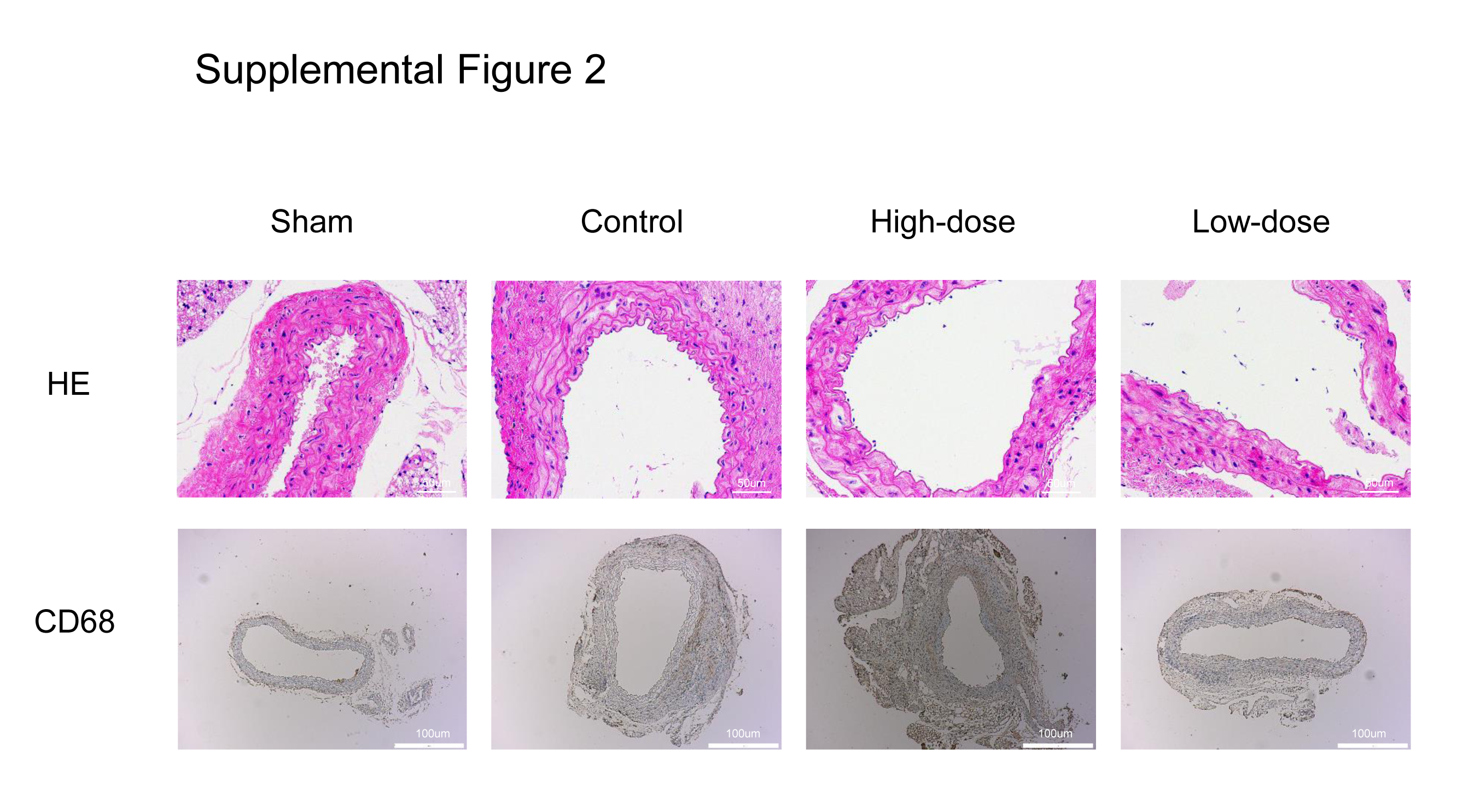

Supplement: Supplementary file 1 [file DataSheet_1.zip › Supplemental material/Supplemental Figure 2.tif]

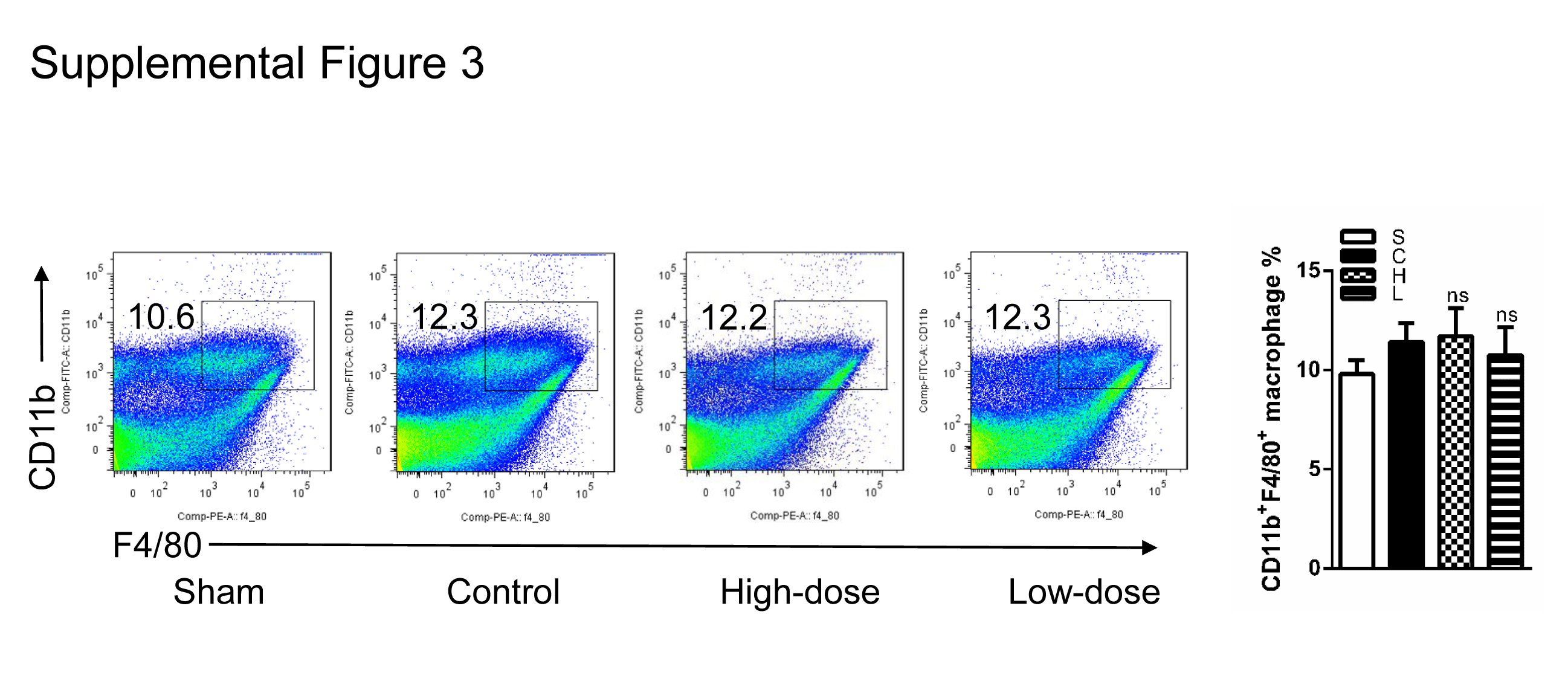

Supplement: Supplementary file 1 [file DataSheet_1.zip › Supplemental material/Supplemental Figure 3.tif]

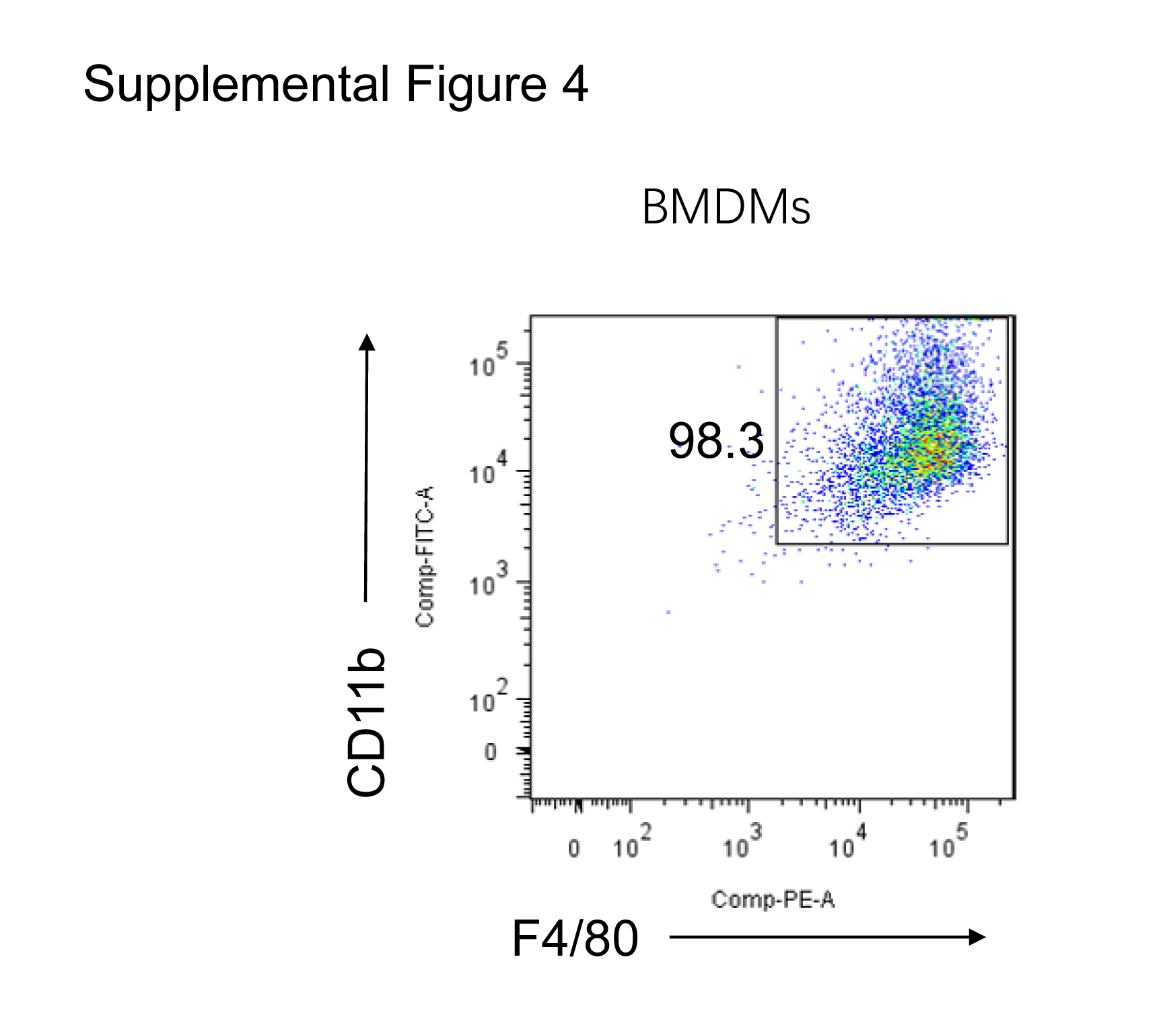

Supplement: Supplementary file 1 [file DataSheet_1.zip › Supplemental material/Supplemental Figure 4.tif]

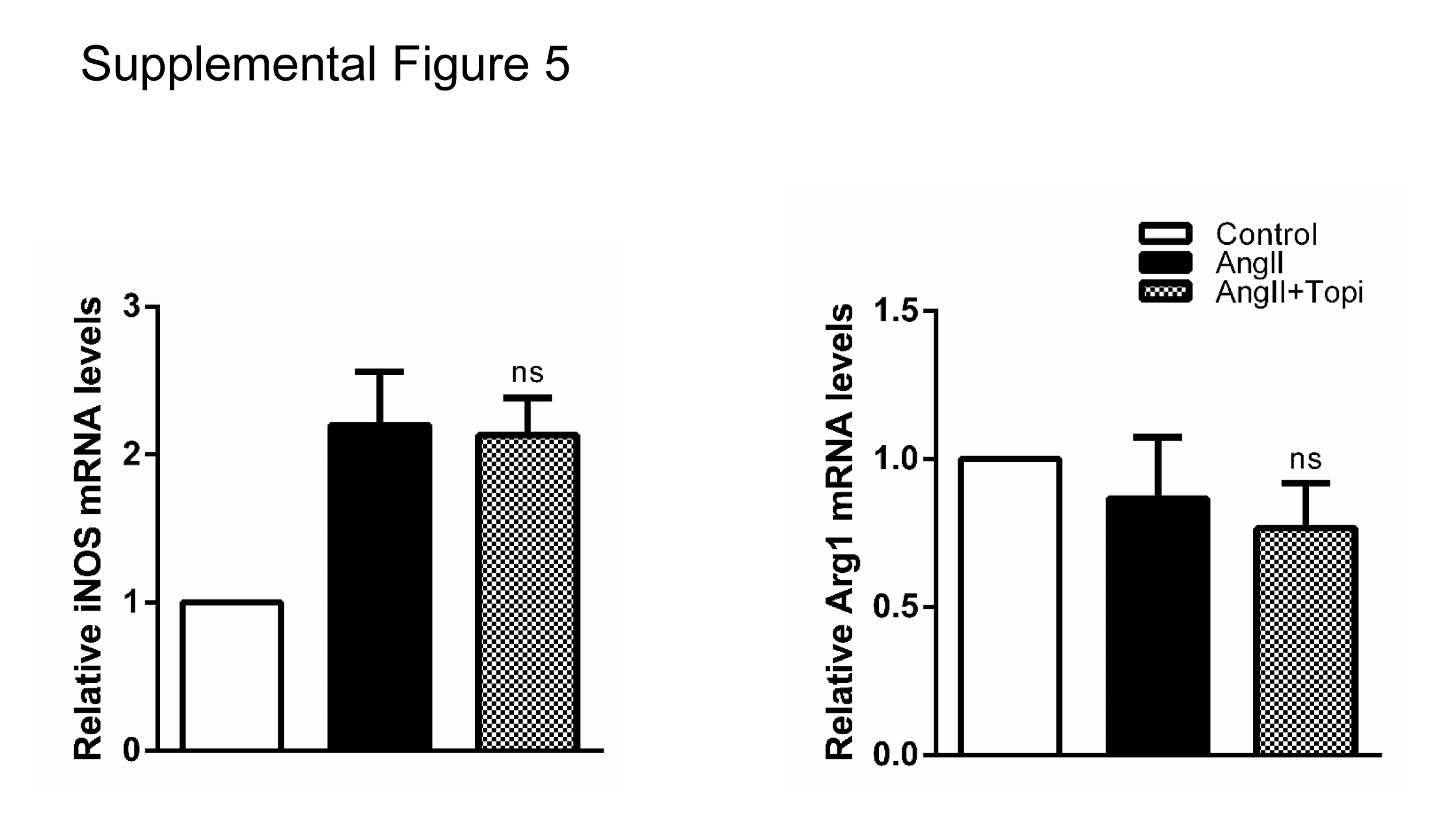

Supplement: Supplementary file 1 [file DataSheet_1.zip › Supplemental material/Supplemental Figure 5.tif]

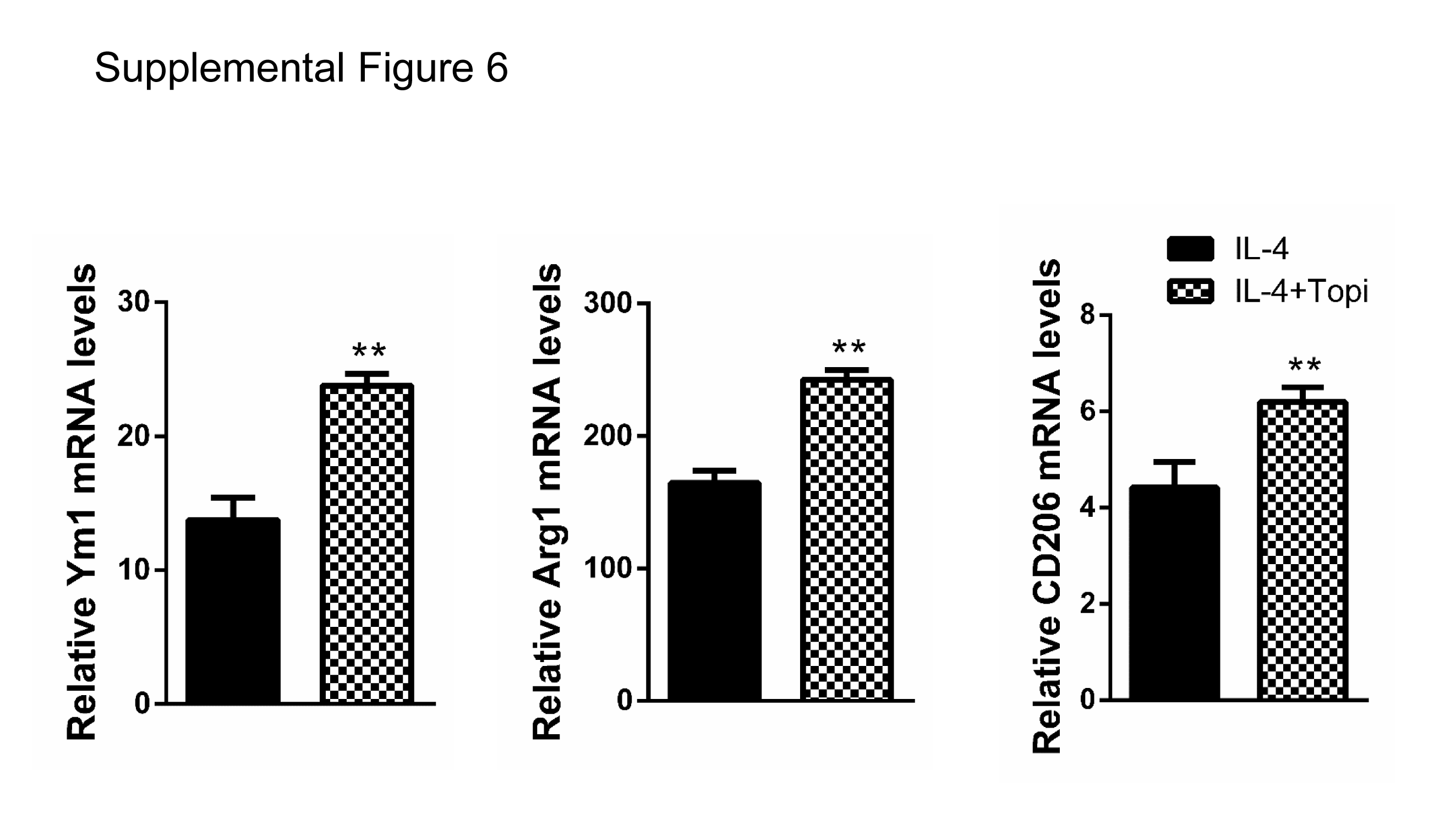

Supplement: Supplementary file 1 [file DataSheet_1.zip › Supplemental material/Supplemental Figure 6.tif]
